# Evaluation of ^198^Au Nanoparticles Coated with Gum Arabic for Prostate Cancer: A Contextual Comparison with BSA-Coated

**DOI:** 10.1021/acsomega.5c01385

**Published:** 2025-07-14

**Authors:** Angélica Bueno Barbezan, Wilmmer Alexander Arcos Rosero, Daniel Perez Vieira, Maria Eduarda Zaganin Rigo, Giovana Dias da Silva, Alex Alves Rodrigues, Luís Fernando de Almeida, Fabio Fernando Alves da Silva, Andy González Rivera, Natanael Gomes da Silva, Emerson Soares Bernardes, Maria Elisa C. M. Rostelato

**Affiliations:** † IPEN/CETER (Instituto de Pesquisas Energéticas e Nucleares/Centro de Tecnologia das Radiações) Av. Prof. Lineu Prestes, 2242 Cidade Universitária, Butantã, São Paulo, SP CEP: 05508-000, Brasil; ‡ USP/IPEN (Universidade de São Paulo—Instituto de Pesquisas Energéticas e Nucleares) Av. Prof. Lineu Prestes, 2242 Cidade Universitária, Butantã, São Paulo, SP CEP: 05508-000, Brasil; § IPEN/CBIO (Instituto de Pesquisas Energéticas e Nucleares/Centro de Biotecnologia) Av. Prof. Lineu Prestes, 2242 Cidade Universitária, Butantã, São Paulo, SP CEP: 05508-000, Brasil; ∥ IPEN/CR (Instituto de Pesquisas Energéticas e Nucleares/Centro de Radiofarmacia) Av. Prof. Lineu Prestes, 2242 Cidade Universitária, Butantã, São Paulo, SP CEP: 05508-000, Brasil

## Abstract

Prostate cancer, a leading cause of morbidity and mortality among men, requires safer and more effective therapeutic approaches. Nanotechnology has emerged as a promising strategy to optimize therapeutic agent distribution and reduce adverse effects of conventional treatments. This study presents the development and evaluation of radioactive gold nanoparticles (^198^AuNPs) coated with gum arabic (GA) for prostate cancer treatment. While BSA-coated nanoparticles have been previously studied by our group, demonstrating therapeutic effectiveness, stability, and biocompatibility, the current work focuses exclusively on the GA formulation, which features a distinct synthesis protocol. *In vitro* and *in vivo* assays with ^198^AuNPs-GA demonstrated good stability, low toxicity in nontumor cells, tumor retention, and reduced tumor growth in animal models. References to BSA are included only for contextual comparison. These findings support the feasibility of gum arabic-coated ^198^AuNPs and offer new insights for enhancing future clinical strategies.

## Graphical abstract

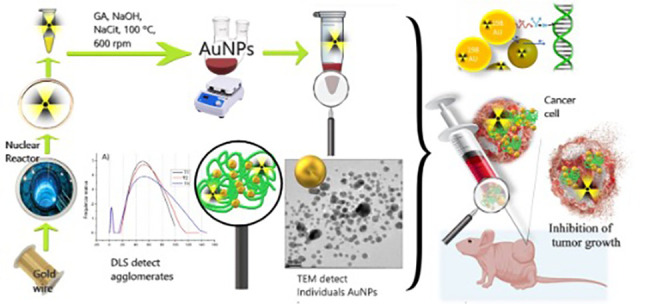

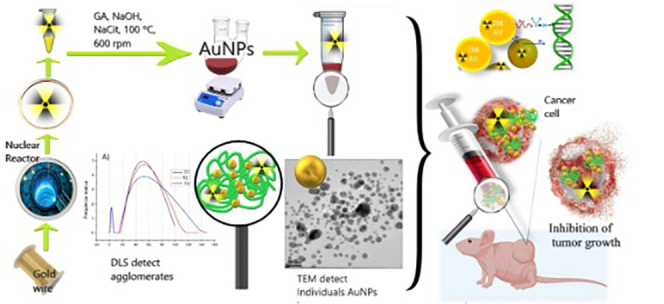

## 1 Introduction

Cancer remains a leading cause of morbidity and mortality worldwide, with its burden continuing to rise due to population aging and lifestyle transitions. Among the various types of cancer, prostate cancer holds particular significance, not only as a common malignancy among men but also due to its potential for significant clinical and socioeconomic impact. Advances in diagnostic techniques, treatment modalities, and public health policies have influenced both its incidence and outcomes, yet disparities in access to care and regional differences in screening practices persist, shaping the global landscape of this disease.

Prostate cancer is the most common type of cancer among men, excluding skin cancer. In 2023, approximately 288,300 men in the United States were projected to be diagnosed with prostate cancer. Globally, the number of prostate cancer diagnoses reached about 1,414,259 cases in 2020, making it the fourth most frequently diagnosed cancer worldwide.^1,2^ There was a notable decline in prostate cancer incidence between 2007 and 2014, mainly attributed to screening guidelines that reduced the use of prostate-specific antigen (PSA) tests. However, since 2014, there has been an annual increase of approximately 3% in global incidence rates, with an even more pronounced increase of 5% per year in advanced prostate cancer cases.^3,4^

Despite these incidence trends, the global mortality rate for prostate cancer varies widely across countries due to disparities in access to early diagnosis and effective treatments. In 2019, based on data analyzed from 89 countries, the average global mortality rate for prostate cancer was approximately 7.6 deaths per 100,000 men.^5^

Current therapeutic modalities for this pathology include chemotherapy, radiotherapy, and surgical procedures.^2^ Brachytherapy, a radiotherapy modality, is an effective treatment option for prostate cancer, involving the placement of radioactive material directly on or near the tumor.^6,7^ With technological advancements, nanobrachytherapy has emerged as an innovative technique in which radioactive nanoparticles are applied directly into the tumor site, ensuring spatial confinement of radiation and reducing exposure to adjacent healthy tissues.^8,9^

The global rise in prostate cancer incidence highlights the need for innovative and minimally invasive therapies. Gold nanoparticles (AuNPs), a well-established platform in nanomedicine, are known for their biocompatibility and precise targeting capabilities. This study explores AuNPs coated with gum arabic (GA) as a stabilizing agent for nanobrachytherapy, providing a direct comparison with bovine serum albumin (BSA)-coated AuNPs from prior research to evaluate their therapeutic properties and efficacy.^10,11^

Nanoparticles, with dimensions ranging from 1 nanometer (nm) to 100 nm, have been increasingly explored in medicine as delivery vehicles for therapeutic agents.^12,13^ At the nanometer scale, the properties of materials can be finely controlled and adjusted, which has significant implications for nuclear medicine.^14,15^ Gold-198 nanoparticles (^198^AuNPs) stand out in this context, not only as vehicles for radionuclides but also for their intrinsic properties that favor medical applications.^16–18^

Radioactive gold nanoparticles offer promising perspectives for cancer therapy, as gold-198 (^198^Au) and gold-199 (^199^Au) possess suitable half-lives and emit β-particles with desirable energy levels (198Au: *t*_1/2_ = 2.7 days, β_max_ = 0.96 MeV; 199Au: *t*_1/2_ = 3.14 days, β_max_ = 0.46 MeV). Additionally, they emit γ-photons, making them applicable for single-photon emission computed tomography (SPECT). In particular, ^198^Au can be readily obtained at high activity levels by neutron irradiation of monoisotopic gold-197 (^197^Au), making it an effective choice for targeted cancer therapies.^19^

To optimize the functionality and biocompatibility of these nanoparticles, coatings such as GA have been employed. GA, a natural resin, is widely used in the pharmaceutical industry due to its biocompatibility and stabilization properties. When applied as a coating, GA provides greater stability and a controlled release profile of the therapeutic or diagnostic agent, enhancing the effectiveness of ^198^AuNPs, especially in therapeutic applications.^20–22^

The extensive scientific literature offers numerous methodologies for synthesizing gold nanoparticles, exploring different geometries such as spheres, stars, and cylinders. However, the field of radioactive nanoparticles remains relatively unexplored, with an evident scarcity of studies. The complexity of incorporating radioactive characteristics into nanoparticles imposes unique technical and logistical challenges, resulting in fewer studies in this area and, thus, greater scientific value for the studies conducted.^23,24^ Handling such radioactive nanoparticles requires specialized skills and facilities, limiting research to locations with adequate infrastructure.

While most existing studies use commercial chloroauric acid as a precursor in gold nanoparticle synthesis, this approach does not align with the goal of generating radioactive nanoparticles through neutron activation in nuclear reactors. In our process, solid gold is initially irradiated in the reactor, followed by dissolution and subsequent synthesis of the nanoparticles. This distinct methodology was specifically developed and adapted for this purpose.^25^

In this context, the development of radioactive gold nanoparticles stands out as a significant contribution in nanotechnology. Research into radioactive ^198^AuNPs, especially when coated with GA, promises significant advances in cancer treatment due to their ability to target radiation more effectively to tumors.^26,27^

This study focuses on the application of ^198^AuNPs coated with GA, exploring their synthesis, characterization, and potential applications in nanobrachytherapy for prostate cancer treatment. In contrast to previous formulations, such as the ^198^AuNPs coated with BSA previously investigated by our group, which demonstrated viability in biomedical contexts, this work examines GA as an alternative stabilizing agent.^28^ We conduct a direct comparison of the therapeutic properties and efficacy of GA and BSA coatings, aiming to identify significant differences and similarities in their biological interactions and therapeutic potentials in the context of oncological treatment.

## 2 Materials and Methods

### 2.1 Synthesis of Gold Nanoparticles with Gum Arabic

The method employed for this synthesis is currently in the process of being patented. Specifically, the gold nanoparticles, referred to here as ^198^AuNPs, were obtained through the neutron activation of solid gold in the EA-R1 nuclear reactor, located at the Instituto de Pesquisas Energéticas e Nucleares (IPEN), as illustrated in Figure 1.

**Figure 1:**
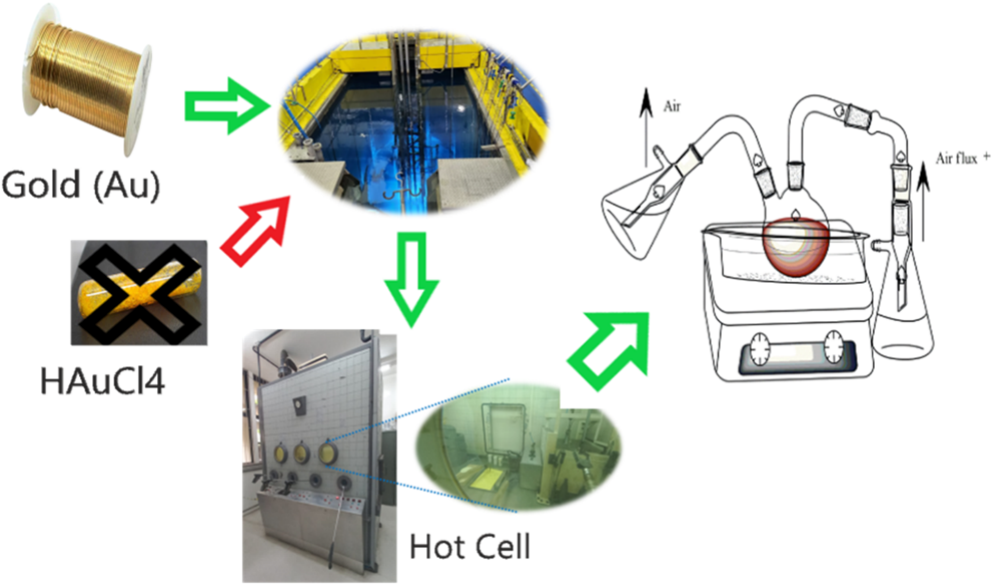
Chemical Reactor for Gold Nanoparticle Synthesis. This reactor is engineered for safe *in situ* synthesis of chloroauric acid and radioactive nanoparticles, featuring air filtration to remove impurities and a system to condense and neutralize acid gases. The process occurs within a specialized hood compliant with Brazil’s National Nuclear Energy Commission and International Atomic Energy Agency standards. To produce gold-198 nanoparticles (^198^Au) *via* neutron activation, pure metallic gold (^197^Au) is required instead of commercial chloroauric acid (HAuCl_4_), which contains nonirradiable ionic gold unsuitable for this purpose. The crossed-out HAuCl_4_ in the figure indicates that this reagent was deliberately excluded from the synthesis.

^198^AuNPs have been the focal point of this study, prepared through a unique process that begins with the neutron activation of solid gold at the EA-R1 nuclear reactor, located at the Instituto de Pesquisas Energéticas e Nucleares (IPEN).

It is important to note that the nanoparticles themselves are not prepared in the reactor; instead, it is the gold that is irradiated. The isotopic purity of the gold, supplied by OUROMINAS, was meticulously analyzed postirradiation using a Hyper Pure Germanium detector. This step was crucial to ensure the successful production of high-quality ^198^AuNPs for further applications in the study.

A variety of reagents played crucial roles in this process: nitric acid (HNO_3_ P.A—Synth), hydrochloric acid (HCl 37% P.A—Synth), sodium hydroxide in the form of pearls (NaOH P.A—Exodo), sodium citrate P.A (CRQ), gum arabic (Synth), polyethylene with a molecular weight of 2k (Laysan Bio). Additionally, the research utilized highly purified water, surpassing the quality of Milli-Q, which was confirmed by the absence of nanometric bodies in Dynamic Light Scattering (DLS) analysis. Ensuring purity and contamination-free conditions, all the glassware and instruments used were thoroughly cleansed with aqua regia and rinsed with nanopure water.

#### 2.1.1 Functionalization of Gold Nanoparticles with Gum Arabic (GA)

GA was used in the synthesis of AuNPs as a stabilizing and coating agent, preventing aggregation and enhancing dispersion, which is crucial for uniformity and biocompatibility in biomedical applications. This stability facilitates the subsequent therapeutic application of AuNPs.

The synthesis process was carried out in a 50 mL round-bottom flask immersed in an oil bath at a controlled temperature of 100 °C, as shown in Figure 1. A mixture of 136.18 μL of *in situ* radioactive chloroauric acid solution (3 × 10^–2^ M), 136.18 μL of GA (5.4 × 10^–5^ M), and 387.19 μL of nanopure water was stirred vigorously at 600 rpm. After 30 s, 10 μL of NaOH (14 M) and 330.45 μL of Sodium Citrate (1 M) were added, leading to the rapid formation of nanoparticles, which were stirred for an additional 3 min before removal from the heat source.

Characterization focused on comparing radioactive and nonradioactive nanoparticles. Nonradioactive AuNPs were analyzed using Dynamic Light Scattering (DLS, Anton Paar Litesizer 500) and Transmission Electron Microscopy (TEM, JEM-2100 Jeol with EDS). Radioactive nanoparticles, due to contamination risks, were characterized solely with DLS. By correlating the results with those of nonradioactive samples, the properties of radioactive AuNPs were reliably inferred. The nanoparticle concentration, calculated through microscopy data and gold mass in the synthesis, was approximately 5.43 × 10^16^ nanoparticles per milliliter.

### 2.2 *In Vitro* Assays—Cytotoxicity Test of GA-Coated AuNPs

The cell lines were selected for their biological characteristics and relevance to prostate cancer. PC-3 and LNCaP represent aggressive and hormone-sensitive models, respectively, while RWPE-1, a noncancerous line, assesses selective toxicity. HUVEC was included to evaluate potential off-target effects. This selection provides a comprehensive assessment of the nanoparticles efficacy and safety.

The cell lines used in this research were sourced from the Rio de Janeiro Cell Bank (BCRJ), including prostate cancer cells (LNCaP/PC3), noncancerous prostate cells (RWPE-1), and umbilical endothelial cells (HUVEC).

The choice of Opti-MEM as the culture medium for the cytotoxicity assays was based on previously validated protocols reported in the literature. Opti-MEM has been successfully used in low-serum or serum-free conditions for cytotoxicity and apoptosis studies, providing consistent results comparable to those obtained with standard culture media, such as DMEM. In a recent study, Lee et al.^29^ demonstrated that Opti-MEM was effective in maintaining cell viability and inducing apoptosis in breast cancer cell lines, validating its applicability for cytotoxicity assays.

Cells were cultivated in 75 cm^2^ culture flasks with Opti-MEM medium (Gibco), supplemented with 5% fetal bovine serum (FBS) and 1% antibiotic-antimycotic solution (penicillin/streptomycin/amphotericin B), and maintained at 37 °C in a 5% CO_2_ incubator. Upon reaching confluence, cells were detached using trypsin/EDTA (0.25%/0.05 M) and seeded in 96-well plates at a density of 5000 cells/100 μL/well. After 24 h of adhesion, the culture medium was replaced with fresh medium containing AuNPs-GA suspensions (radioactive or nonradioactive) or toxicity controls (10% NaCl as negative control; 10% DMSO as positive control), diluted at concentrations ranging from 2.5 to 20 μL per 100 μL/well.

All *in vitro* cytotoxicity assays were performed in octuplicate (eight technical replicates per condition) within the same plate, with control and treatment groups distributed in separate wells to ensure uniform conditions. The final experiment was performed only once, as a consolidated assay including all tested cell lines. During the development of the study, preliminary individual evaluations were conducted with each cell line under equivalent conditions, and since no significant differences were observed between the results, the simultaneous protocol was adopted to optimize experimental consistency. Due to force majeure circumstances at the institution, including limited availability of the radioactive isotope (^198^Au) and operational constraints, it was not possible to perform additional biological replicates at this stage. Nevertheless, the study was conducted under strictly controlled conditions and is presented as a proof-of-concept. Future studies should include independent biological replicates to further validate and deepen the findings. Cells were incubated with the nanoparticle suspensions or controls for 6, 24, or 48 h.

After incubation, cells were washed with Dulbecco’s Phosphate-Buffered Saline (dPBS) to remove free particles. Next, 20 μL of [3-(4,5-dimethylthiazol-2-yl)-5-(3-carboxymethoxyphenyl)-2-(4-sulfophenyl)-2*H*-tetrazolium]/Phenazine methosulfate (MTS/PMS) solution, prepared following the manufacturer’s instructions (CellTiter 96 AQueous Non-Radioactive Cell Proliferation Assay), was added to each well, diluted in 100 μL of fresh culture medium. Absorbance was measured at 490 nm using a Multiskan EX plate reader (Thermo Scientific) after a 2–4-h incubation period.

### 2.3 *In Vivo* Assays—Treatment with GA-Coated ^198^AuNPs

For this study, the BALB/C Nude mice breed was selected, sourced from the animal facility at IPEN, adhering to protocols approved by the CEUA (Ethics Committee on the Use of Animals), protocol number 243/19.

The *in vivo* experiments were conducted in accordance with the ARRIVE 2.0 (Animal Research: Reporting of *In Vivo* Experiments) guidelines, adapted to the scope of the project and the protocol approved by the institutional animal ethics committee (CEUA). The number of animals used was defined based on criteria of animal use reduction and logistical limitations related to handling radioactive material, in alignment with the 3Rs principles (Replacement, Reduction, and Refinement).

Mice, specifically male, aged 8 to 12 weeks and averaging around 25 g in body weight, were chosen. This choice was made to ensure consistency in the biological response and to limit variables that are not directly related to the experimental treatments.

Regarding the tumor cell line and inoculation process, the PC-3 line was employed. The mice received subcutaneous injections of 5 × 10^6^ PC-3 cells into the upper right hind paw area, with a total volume of 100 μL. This solution was a 50% blend of the extracellular matrix (Gibco Geltrex LDEV-Free, hESC-Qualified, Reduced Growth Factor Basement Membrane Matrix).

Group 1 (*n* = 8): This group was treated with radioactive gold nanoparticles (^198^AuNPs GA) at an average activity level of 600 μCi (22,2 Mbq).

Control Group (*n* = 6): This group did not receive any radioactive treatment.

Biodistribution Study: Biodistribution Study: 2 animals from group 1 were selected to evaluate the biodistribution of ^198^AuNPs GA at two moments, specifically 3 and 24 h after administration.

^198^AuNPs GA Administration: The mice were anesthetized using inhaled anesthesia (Isoflurane—Cristália), followed by the intratumorally administration of a 30 μL aliquot per animal.

Tumor Monitoring: After administration of the ^198^AuNPs GA, tumor volumes were measured twice a week over a period of 21 days using a digital caliper.

Housing conditions: The rodents were kept in specialized cages, under a controlled environment, with unrestricted access to water and food, complying with recommended care standards.

#### 2.3.1 Biodistribution Studies of GA-Coated ^198^AuNPs in Animal Models

Following the previously described protocol regarding the evaluation of the therapeutic efficacy of AuNPs GA, the animals were prepared in an identical way.

After the administration of the nanoparticles, two distinct time points were established for the biodistribution analysis: 3 and 24 h postinjection. Accordingly, two animals were designated—one for each time point. The number of animals used in the biodistribution study was predetermined and approved by the CEUA (Comitê de Ética no Uso de Animais), under protocol number 243/19. This approval defined a restricted number of animals, with specific allocation for each type of analysis proposed in this preliminary investigation.

During the stipulated analysis interval, the animals were anesthetized with isoflurane and a 30 μL blood sample was collected using the retro-orbital method. The harvested organs (Blood, Heart, Lung, Liver, Kidneys, Gallbladder, Spleen, Stomach, Small Intestine, Large Intestine, Pancreas, Bones, Muscles, Brain, Fat, Bladder and Tumor) stored in labeled tubes and subsequently analyzed quantitatively with a γ Counter (γ-2470 Automatic γ Counter from PerkinElmer) to evaluate the biodistribution of nanoparticles (%ID/g - Injected dose per g).

#### 2.3.2 Evaluation of Hematological Parameters Following Treatment with ^198^AuNPs GA

Following the treatment with GA coated AuNPs, a detailed hematological examination was conducted on a group of four mice to assess their blood health. This examination took place after all therapeutic efficacy tests were completed and just before euthanasia. For the procedure, the mice were anesthetized using isoflurane and approximately 300 μL of blood was collected from each animal. After the collection, the mice were then euthanized. The blood samples were carefully transferred to appropriately labeled tubes, which already contained EDTA as an anticoagulant, ensuring proper preservation of the samples for subsequent analyses.

These samples were then dispatched to LAB&VET Diagnostic and Veterinary Consultancy for comprehensive hematological examination. The differential leukocyte count was carried out using the ABC PENTRA 80 system, a product of HORIBA. The final report of the analysis was officially certified and authenticated by Dr. Naiadi A. Publio, bearing the CRMV-SP license number 32904.

## 3 Statistical Analyses

Statistical analyses were performed using GraphPad Prism 9.5.0 (GraphPad Software). For *in vitro* experiments (*n* = 8), one-way ANOVA followed by Bonferroni’s post hoc test was applied. For *in vivo* studies (*n* = 6 per group), two-way repeated measures ANOVA with Bonferroni correction was used. Data distribution was assessed using the Shapiro–Wilk normality test and confirmed to follow a Gaussian distribution (*p* > 0.05 for all groups). Statistical significance was considered at *p* < 0.05 (*p < 0.05; **p < 0.01; ***p < 0.001; ****p < 0.0001). Raw data from equipment were organized using Microsoft Excel version 16.76 prior to analysis. Characterization graphs (ultraviolet–visible (UV–vis), DLS) were generated using Origin 9.0 (OriginLab).

## 4 Results

### 4.1 Synthesis of AuNPs GA

The characterization of radioactive gold nanoparticles (^198^AuNPs) focused on evaluating their morphology, size distribution, and stability. Figure 2 presents the characterization results, where image A shows the Transmission Electron Microscopy (TEM) analysis confirming that the synthesis process produced nanoparticles with a consistent spherical morphology and relatively uniform size distribution. Graph B displays the particle size distribution profiles of the ^198^AuNPs before treatment, demonstrating a predominant size range between approximately 40 and 100 nm. Graph C shows the distribution profiles after treatment, indicating that the GA coating effectively maintained the structural stability of the ^198^AuNPs, with no significant aggregation observed. These findings provide essential information on the physicochemical properties of ^198^AuNPs, supporting their suitability for subsequent biological evaluations.

**Figure 2:**
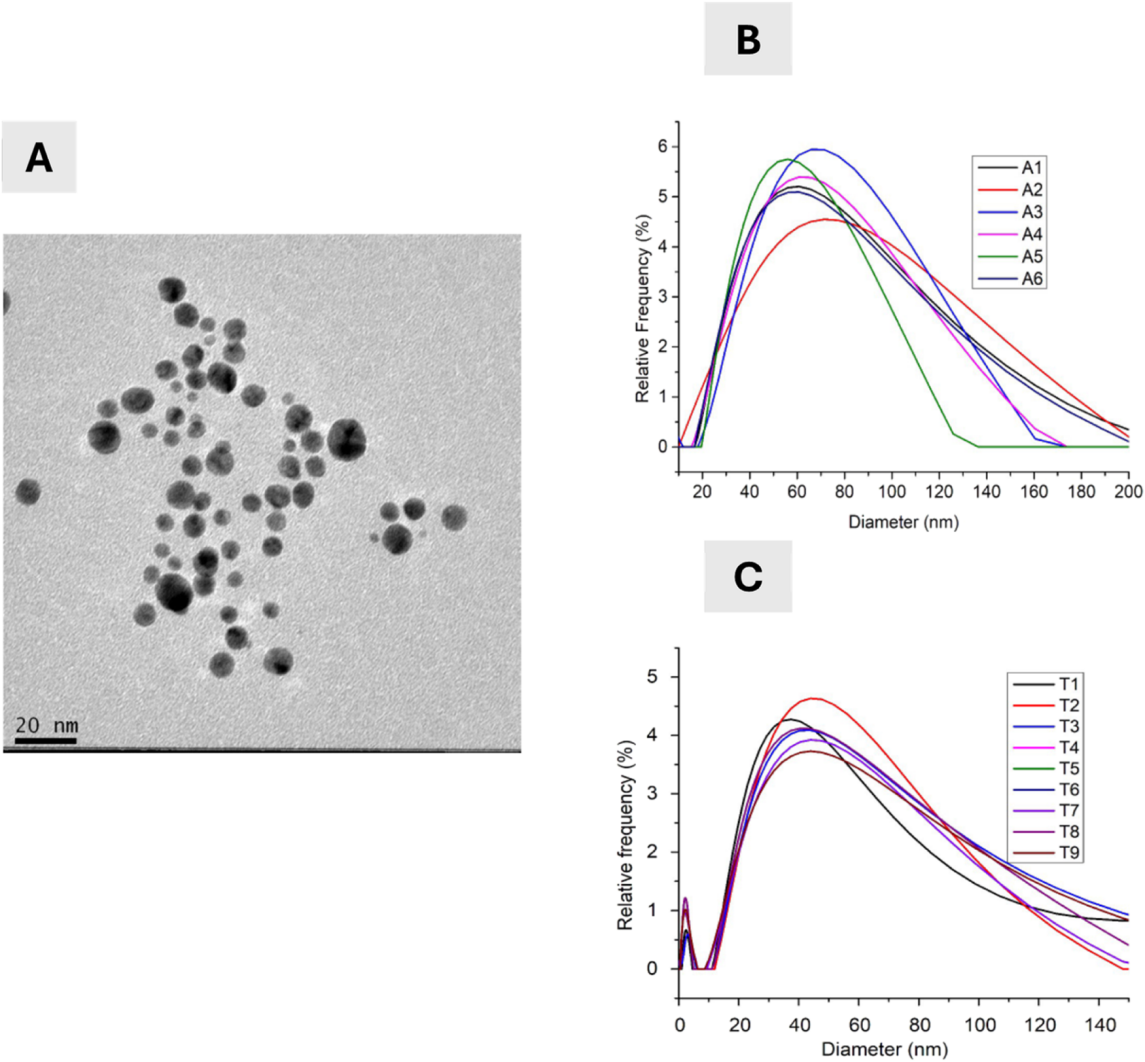
Comprehensive characterization of AuNPs GA. (A) Transmission Electron Microscopy (TEM) image of AuNPs GA, showing a uniform spherical morphology with an average core size of approximately 5 nm. (B) Particle size distribution profiles of different synthesized samples (A1–A6) before treatment, demonstrating reproducibility and size uniformity. (C) Particle size distribution profiles of samples (T1–T9) after treatment, confirming the stability and structural integrity of the nanoparticles.

Dynamic Light Scattering (DLS) analyses in our study indicate that gum arabic-coated nanoparticles in aqueous solution tend to form aggregates with an average hydrodynamic diameter of approximately 50 nm. Despite this aggregation, the GA coating effectively stabilizes the nanoparticle core, maintaining a size of around 5 nm. Due to facility constraints, DLS was the only feasible method for characterizing radioactive nanoparticles, providing essential insights into their size distribution.

Comparative DLS measurements further confirmed the average size consistency of the radioactive nanoparticles. These findings provided the foundation for subsequent *in vitro* and *in vivo* investigations to evaluate their therapeutic potential.

To estimate the nanoparticle concentration, the total number of atoms was divided by the number of atoms per nanoparticle. For particles with a diameter of 5.32 nm, the volume was calculated using the sphere formula: *V* = 4/3π*r* ^3^ = 78.838 nm^3^. Given a gold atom radius of 0.166 nm and an atomic volume of approximately 0.70 nm^3^, the estimated number of atoms per nanoparticle was 78.838/0.70 ≈ 112.63 atoms. Image analysis of TEM micrographs was performed using the ImageJ software, and statistical treatment was conducted in Origin 9.0.

To confirm the gold concentration in the nanoparticle suspension, Inductively Coupled Plasma Optical Emission Spectrometry (ICP-OES) was performed after sample digestion in aqua regia. The analysis, conducted in duplicate, yielded an average gold concentration of 648.27 ppm, slightly lower than the theoretical 804.70 ppm, based on the initial HAuCl_4_ concentration (0.03 M). This deviation is attributed to material loss during the synthesis process, particularly due to vapor stripping.

The ICP-OES analysis was conducted using a Spectro Arcos instrument (radial view). Calibration was carried out with certified gold standard solutions (1000 mg/L) diluted in aqua regia, covering a range of 0.1 to 10.0 mg/L. The analytical line used was Au I 242.795 nm. The calibration curve showed excellent linearity (*R* ^2^ ≥ 0.999), and an intermediate quality control standard (2.5 mg/L) was included for validation. Background correction was applied, and all sample dilutions were prepared in the same matrix as the standards. Instrument stability was monitored by reanalyzing a calibration standard every ten samples.

ζ-potential analysis and time-dependent particle size distribution are shown in Figure 3 (images A and B, respectively). A peak near 0 mV would suggest colloidal instability; however, no significant aggregation was observed throughout the monitoring period.

**Figure 3:**
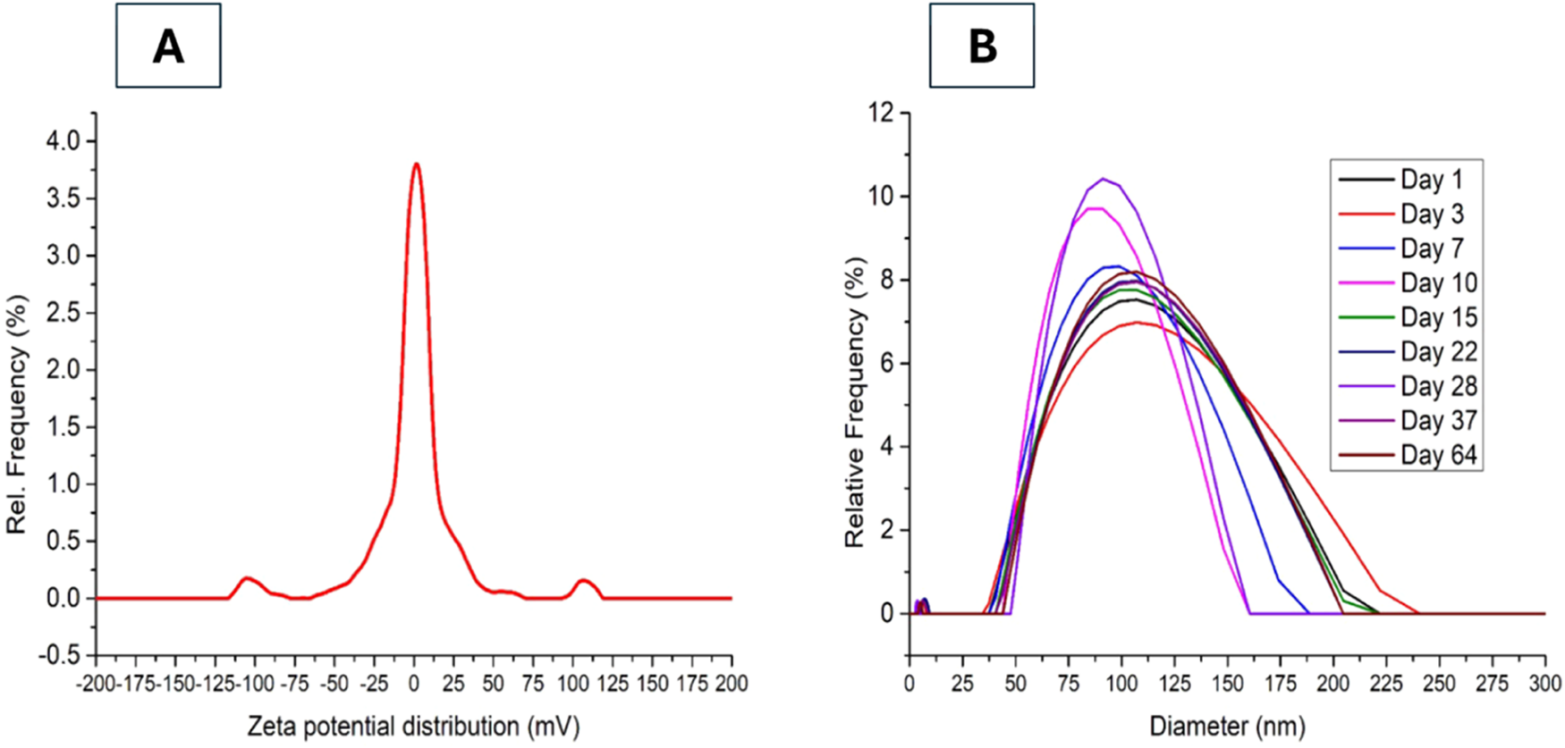
Characterization of the stability of GA-coated ^198^AuNPs. (A) ζ-Potential distribution analyzed by Dynamic Light Scattering (DLS), showing a distinct peak far from 0 mV, which indicates good colloidal stability. (B) Time-dependent particle size distribution, demonstrating the maintenance of nanoparticle stability over different storage periods, as measured by DLS.

Regarding long-term stability, a radioactive sample was monitored over time. Figure 3 presents the stability assessment of GA-coated ^198^AuNPs over a 64-day period, considering the half-life of *t*_1/2_ = 2.7 days. The ζ-potential analysis, shown in image A, demonstrates that the nanoparticles maintained an adequate surface charge, indicative of good colloidal stability. After approximately 28 days (corresponding to ten half-lives), the stability of the nanoparticles becomes less critical. By this time, the gold nanoparticles are expected to have sufficiently decayed, no longer posing a radiological risk.

As a result, potential disintegration, agglomeration, or even clearance from the tumor possibly due to tumor shrinkage could lead to their natural elimination by the immune system, as they would be recognized as foreign bodies. The particle size distribution over time, illustrated in image B, shows minor variations in the size of gold nanoparticle agglomerates throughout the 64-day period, further confirming their stability.

### 4.2 Assessment of the Cytotoxic Effects of Non-Radioactive GA-Coated AuNPs

To support the evaluation of cytotoxicity in various cell lines, Table 1 summarizes the concentrations and volumes of AuNPs GA tested *in vitro*.

**Table 1:** Concentrations and Volumes (in μL) of AuNPs GA Used in Cytotoxicity Assays with LNCaP, PC-3, RWPE-1, and HUVEC Cell Lines

volume (μL)	Au (mg)	concentration AuNPs GA
20	0.013	5.55 × 10^11^
17.5	0.011	4.70 × 10^11^
15	0.0097	4.15 × 10^11^
12.5	0.0081	3.47 × 10^11^
10	0.0065	2.78 × 10^11^
7.5	0.0049	2.07 × 10^11^
5	0.0032	1.37 × 10^11^
2.5	0.0016	6.84 × 10^10^

In this study, we adapted our methodological approach to investigating the cytotoxicity of AuNPs GA in various cell lines. Recognizing the importance of exploring the long-term effects of AuNPs exposure and aiming to enhance our temporal analysis of cytotoxicity, we opted to adjust the evaluation intervals to 24, 48, and 72 h. This modification allows us to capture a more comprehensive view of the cellular response dynamics over time, as potential cytotoxic effects may not manifest in the first few hours after exposure. The nanoparticle concentrations remained consistent with the previous study conducted by the group, ranging from 2.5 to 20 μL, to allow for a direct comparison, while at the same time expanding our understanding of AuNPs behavior over an extended period. The results of this study are presented in Figure 4.

**Figure 4:**
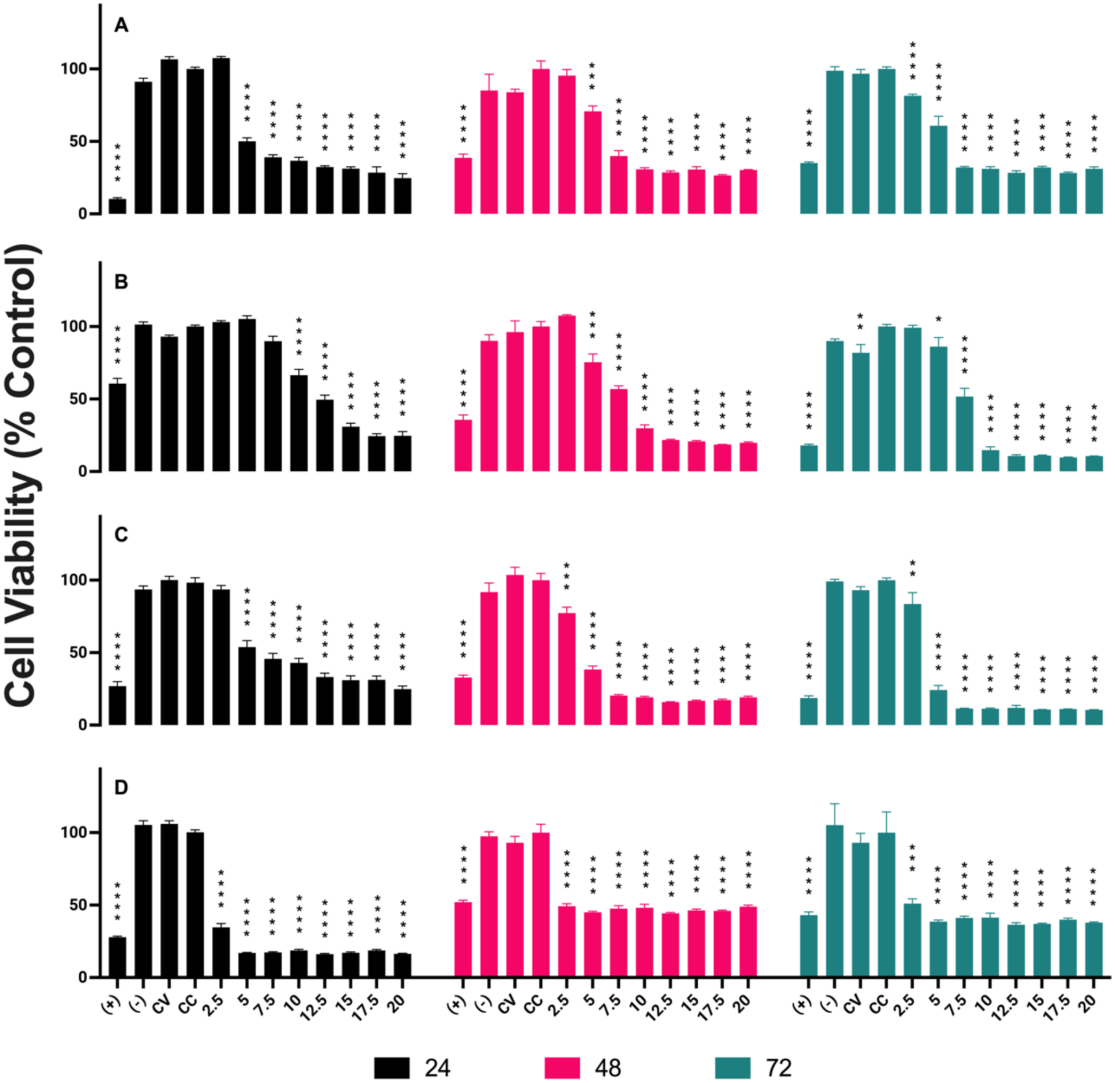
Cytotoxic Response of Non-Radioactive GA-Coated AuNPs in Different Cell Lines. The cytotoxic effects of nonradioactive GA-coated gold nanoparticles (AuNPs GA) were evaluated in four cell lines: (A) PC-3 (prostate cancer), (B) LNCaP (prostate cancer), (C) RWPE-1 (noncancerous prostate cells), and (D) HUVEC (human umbilical vein endothelial cells). The experiments were conducted over three exposure durations: 24 h (black bars), 48 h (pink bars), and 72 h (green bars). Cell viability was assessed relative to untreated controls, showing a progressive decrease with increasing AuNPs GA concentrations across all cell lines. The cytotoxic effects were both dose- and time-dependent, with statistically significant differences from the untreated control indicated by *p < 0.05, **p < 0.01, ***p < 0.001, ****p < 0.0001.

For the PC3 cell line (Graph A), we observed a progressive decrease in cell viability with increasing concentration of AuNPs GA and exposure time. Our study revealed a significant cytotoxic response at all tested concentrations, consistent with the findings of Barbezan et al.^28^

In the LNCaP cell line (Graph B), we also observed a dose-dependent cytotoxic response to AuNPs GA, with a reduction in cell viability compared to the control. These results are consistent with the findings of the previous study, highlighting the efficacy of AuNPs GA as potential cytotoxic agents.

The graphs for the RWPE-1 (Graph C) and HUVEC (Graph D) cell lines display similar patterns of cytotoxic response to AuNPs GA over time. Cell viability decreased progressively with increasing nanoparticle concentration and exposure time, indicating a consistent and dose-dependent cytotoxic effect. These results corroborate the observations made by Barbezan et al.^28^ in their study with AuNPs BSA.

In summary, our results suggest that AuNPs GA have potential as cytotoxic agents in a variety of cell lines, with response patterns similar to those observed with AuNPs BSA in the previous study. This similarity highlights the versatility of both coatings, demonstrating comparable efficacy while allowing flexibility in selecting coatings based on other properties, such as biocompatibility or stability, for therapeutic applications.

After contextualizing the study by Barbezan et al.,^28^ which served as a comparative basis for our work, we now focus on the analysis of the IC50 values of gold nanoparticles coated with Gum Arabic Figure 5. It was observed, in the cell lines PC3, LNCaP, RWPE- 1 and HUVEC, a dose-dependent cytotoxic response to AuNPs GA treatment. The inclusion of a 72-h incubation period in this study revealed a progressive decrease in cell viability over time, highlighting the cumulative cytotoxicity of the AuNPs GA. This in-depth analysis, in contrast to the shorter periods of the previous study, underlines the importance of somewhat longer time intervals for a comprehensive assessment of cytotoxic effects. The increased IC50 values over 24- and 48-h periods, and the new addition of 72-h data, provide valuable insights into the dynamics of nanoparticle-cell interaction, reinforcing the need to consider temporal response in biomedical applications.

**Figure 5:**
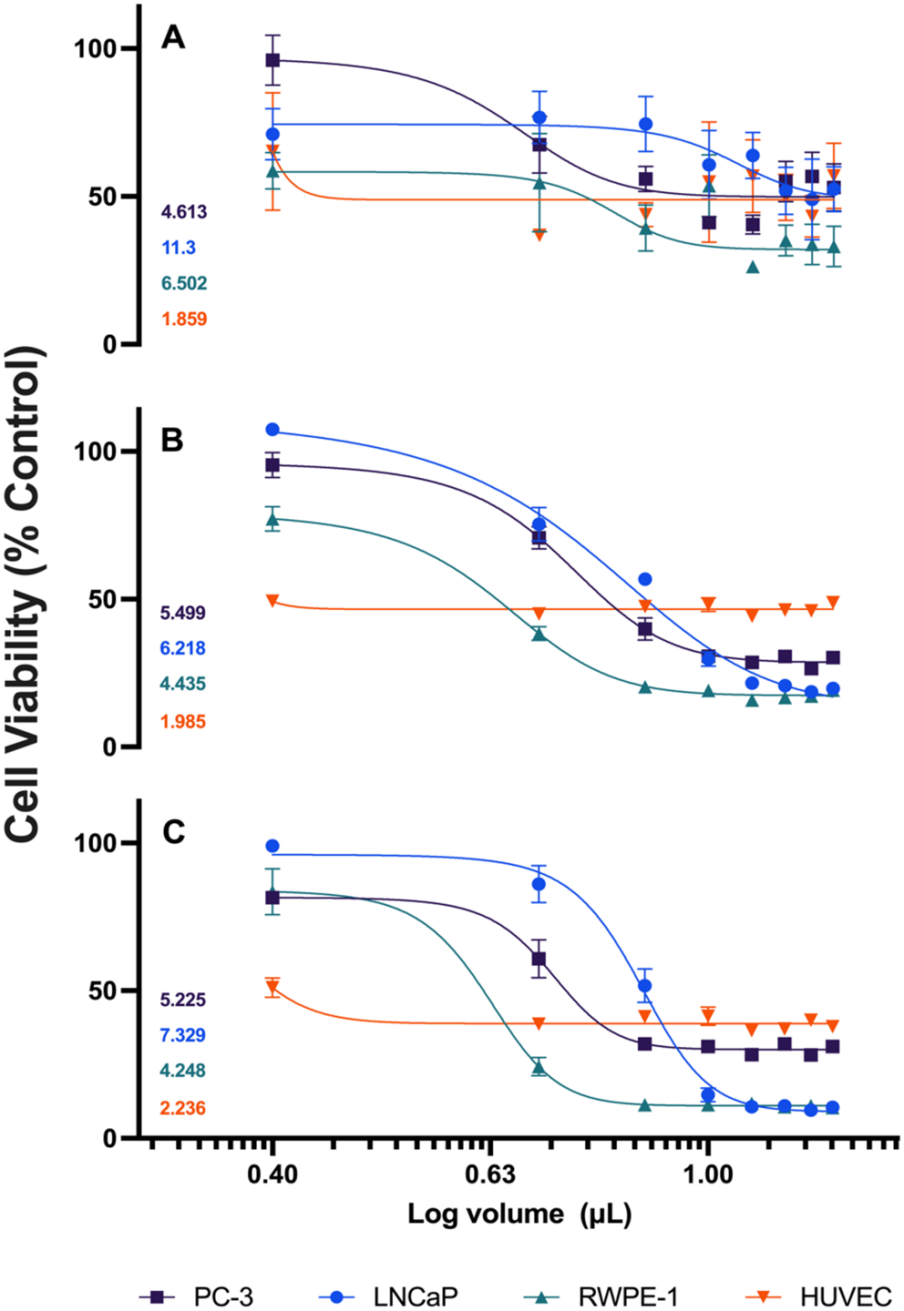
Determination of IC50 values for AuNPs GA in different cell lines over 24, 48, and 72 h of treatment. (A) represents data after 24 h of exposure, (B) after 48 h and (C) after 72 h. The curves show the percentage of cell viability relative to the untreated control for the PC-3 (black lines), LNCaP (blue lines), RWPE-1 (green lines) and HUVEC (red lines) cell lines. The corresponding IC50 values are indicated in each panel. Data are expressed as mean ± standard deviation of the mean (SEM) of three independent biological assays.

### 4.3 Evaluation of the Cytotoxicity of GA-Coated Radioactive Gold Nanoparticles (^198^AuNPs GA)

This study extends to include the cytotoxic effects of radioactive nanoparticles, using the same cellular models as with the nonradioactive counterparts. Rigorous methodology was imperative to maintain the integrity of the experimental outcomes. A dedicated microtiter plate for controls including Positive Control (DMSO), Negative Control (NaCl), and Cell Control (CC) was employed to negate any confounding effects of cross-irradiation from the treated wells.

In the treatment assays, three specific concentrations of ^198^AuNPs GA: 2.5 μL, 5 μL, and 7.5 μL were tested, corresponding to radioactivities of 0.8 μCi, 1.6 μCi, and 2.4 μCi, respectively. Wells were strategically placed to minimize any potential impact from adjacent radiation, thus fostering more reliable cytotoxicity measurements.

Results from the 6-h antiproliferative assays using ^198^AuNPs GA are portrayed in Figure 6. In the LNCaP cell line (Black bars), cell viability reached 131% at 0.9 μCi, indicating a stimulatory effect rather than an antiproliferative one at this concentration. At 1.8 μCi, viability normalized to 102%, and at 2.7 μCi, a modest decline to 91% is observed, suggesting the onset of antiproliferative effects at higher radioactivity levels. In PC-3 cells (Pink bars), stability in viability is seen at 100% compared to control at 0.9 μCi. An increase to 105% is noted at 1.8 μCi, possibly due to a radio-stimulatory effect, while a drop in viability to 76% at 2.7 μCi indicates a clear antiproliferative effect at this elevated radioactivity.

**Figure 6:**
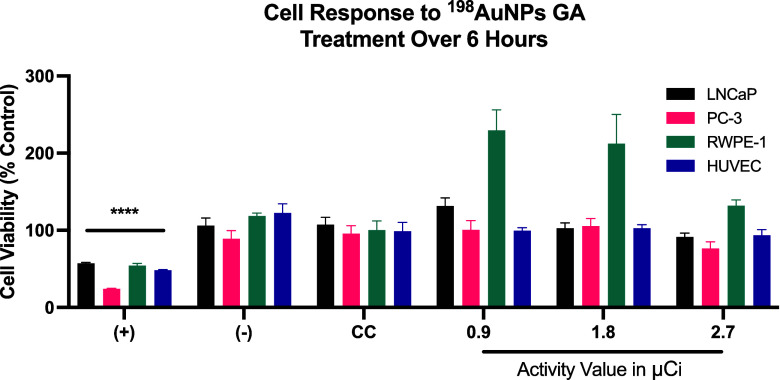
Cytotoxicity Assay of ^198^AuNPs GA in LNCaP, PC-3, RWPE-1, and HUVEC Cell Lines After 6-h Treatment. Black bars shows the response of the LNCaP cell line, indicating an initial stimulatory effect followed by the onset of cytotoxicity at higher radioactivity levels. Pink bars illustrates the impact on PC-3 cells, with stability in viability and signs of cytotoxicity at increased radioactivity. Green bars highlights marked cellular proliferation in RWPE-1 cells at all tested levels. Purple bars portrays the response in HUVEC cells, showing relatively consistent viability across the tested range of radioactivity.

RWPE-1 cells (Green bars) showed marked cellular proliferation with 229% viability at 0.9 μCi, decreasing to 212% at 1.8 μCi, and further to 132% at 2.7 μCi, still showing proliferative behavior at these activity levels. HUVEC cells (Purple bars) exhibited viability at 99% at 0.9 μCi, slightly above the control at 102% for 1.8 μCi, and a slight decrease to 93% at 2.7 μCi, indicating relatively consistent viability across the tested radioactivity range.

Figure 7 presents the assessment of cell viability over a 24-h treatment period, maintaining the same radioactive activities and cell lines previously evaluated. In the LNCaP cell line (black bars), exposure to 0.9 μCi resulted in a marked increase in viability, reaching approximately 130%, suggesting a potential proliferative effect induced by low-dose radioactivity. At 1.8 μCi, viability is reduced to 84%, and further decreases to 68% at 2.7 μCi, demonstrating a clear dose-dependent cytotoxic effect. In PC-3 cells (Pink bars), an initial dose of 0.9 μCi results in a viability of 109% compared to the control, indicating a slight proliferative response. Viability decreases to 91% at 1.8 μCi and further to 72% at 2.7 μCi, showing a consistent decline as radioactivity increases.

**Figure 7:**
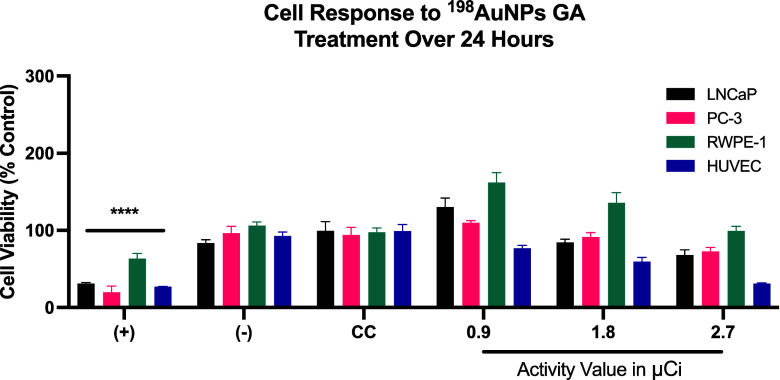
Response of Cell Lines to ^198^AuNPs GA Treatment Over 24 h. Black line shows an initial increase followed by a dose-dependent reduction in viability in LNCaP cells. Pink line reveals a mild initial response and subsequent decline in PC-3 cell viability as radioactivity increases. Green line highlights a significant increase followed by normalization in RWPE-1 cell proliferation at higher doses. Purple line illustrates heightened sensitivity and increasing cytotoxic impact in HUVEC cells in response to rising radioactivity levels.

In RWPE-1 cells (Green bars), a significant increase in cell proliferation to 162% is observed at a 0.9 μCi dose. A reduced yet still heightened proliferation rate of 135% is seen at 1.8 μCi, and at 2.7 μCi, the rate normalizes to 99%, indicating a tapering of the proliferative effect at higher doses. In HUVEC cells (Green bars), a stark decrease in viability to 76% is noted at just 0.9 μCi, suggesting high sensitivity to the radioactive treatment. The viability further drops to 59% at 1.8 μCi and plummets to 31% at 2.7 μCi, highlighting the significant cytotoxic impact of ^198^AuNPs GA at increasing levels of radioactivity.

Conducting a comparative study between the two works, both studies investigated the cytotoxic effects of radioactive gold nanoparticles on the same cell lines, adopting rigorous methodologies to minimize interferences and employing suitable controls (DMSO, NaCl, CC) to ensure the integrity of the experimental results. A notable methodological difference was the approach to mitigating irradiation interference between wells, though both employed strategies to minimize the impact of adjacent radiation.

The results of the current study with AuNPs GA demonstrated a stimulatory effect at specific concentrations and in certain cell lines, with an increase in viability under particular conditions. Interestingly, these findings revealed marked cellular proliferation under certain circumstances, highlighting the complexity of the effects induced by radioactive nanoparticles. This suggests that the biological properties of AuNPs GA are influenced by their coating, which can elicit both cytotoxic and stimulatory responses depending on the dose and the type of cell.

### 4.4 *In Vivo* Assays

#### 4.4.1 Therapeutic Effectiveness of ^198^AuNPs GA

In our previous pilot study (de Souza et al.^25^), we demonstrated that treatment with ^198^AuNPs GA reduced tumor growth in animal models compared to untreated controls, although tumor regression was not observed at that stage. Building on these findings, we conducted a more detailed *in vivo* analysis with an expanded cohort of mice (*n* = 6), administering an average activity of 635 μCi (23.5 MBq) of ^198^AuNPs GA per animal and monitoring tumor development over a 21-day period.

In this study, we compared tumor volume progression over time in two experimental groups: a treated group and a control group. Figure 8 illustrates the results of this comparison, highlighting a statistically significant reduction in tumor volume in the treated group compared to the control (*p* < 0.05), starting from Day 7 and persisting through Days 14 and 21.

**Figure 8:**
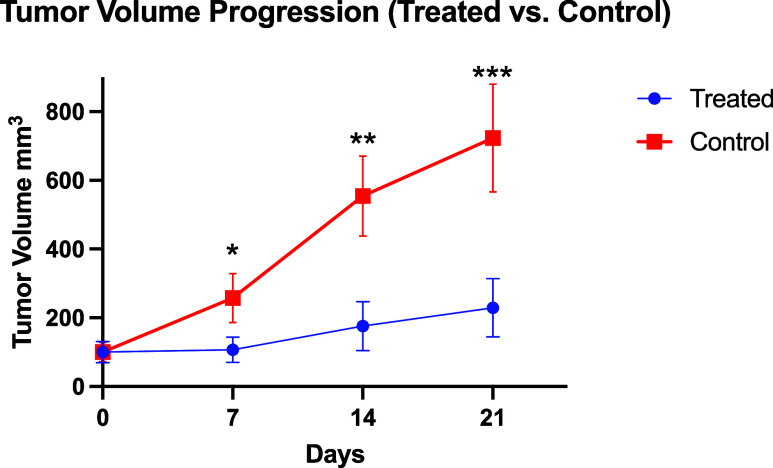
Tumor Growth Dynamics Over Time in Treated and Control Groups. This graph illustrates the progression of tumor volume (mm^3^) over time in two groups of mice: those treated with ^198^AuNPs-GA (blue line) and the untreated control group (red line). Tumor volumes were measured on days 0, 7, 14, and 21 post-treatment. Error bars represent the standard error of the mean (SEM) for each group (*n* = 6). Statistical analysis was performed using two-way repeated measures ANOVA followed by Bonferroni’s posthoc test. Significant differences between treated and control groups are indicated by *p < 0.05, **p < 0.01, ***p < 0.001.

Interestingly, while most treated animals displayed reduced tumor growth rates compared to the control group, one treated mouse exhibited complete tumor regression. Figure 9 presents the tumor volume trajectory of this individual animal over the experimental period.

**Figure 9:**
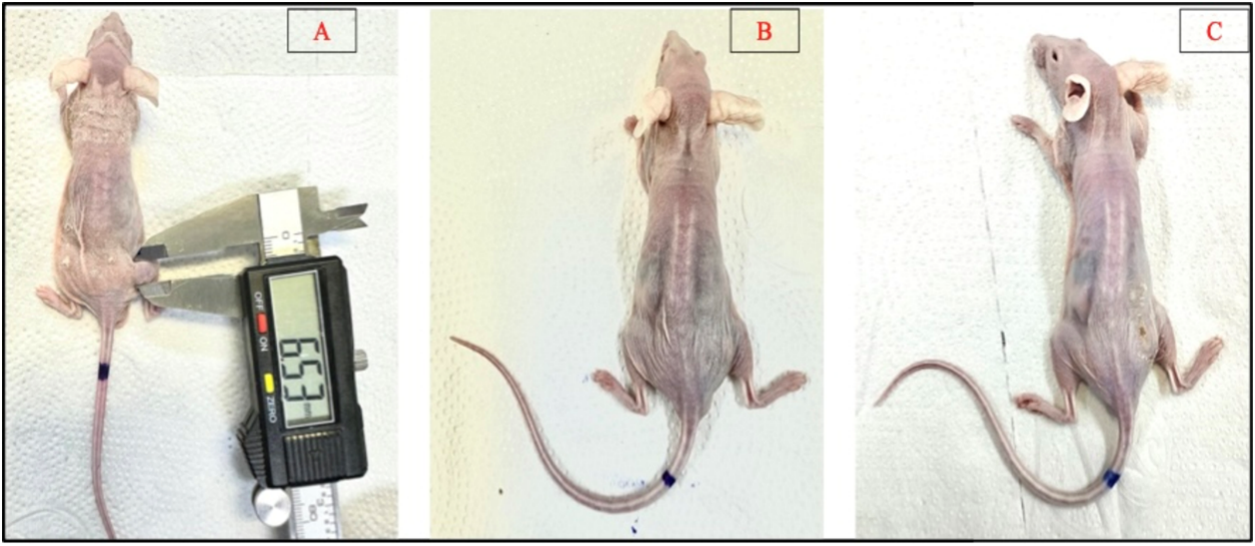
Therapeutic Response to ^198^AuNPs GA in Animal 1. This figure illustrates the progression of tumor volume in Animal 1 after administration of ^198^AuNPs GA with an activity of 635 μCi (23.5 MBq). Panel A shows the initial tumor volume at day 0, serving as a baseline measurement prior to treatment. Panel B displays the absence of detectable tumor volume at day 14 post-treatment, indicating a substantial therapeutic effect. Panel C, corresponding to day 21 post-treatment, demonstrates a continued absence of tumor volume, highlighting the sustained efficacy of ^198^AuNPs GA in reducing tumor burden over time.

Overall, the treated group consistently demonstrated slower tumor growth compared to the control, with significant differences observed throughout the study period. These results provide robust evidence of the therapeutic efficacy of ^198^AuNPs GA and justify further investigations with larger sample sizes and complementary analyses to validate and expand these promising initial findings.

A significant reduction in tumor volume was observed in the animals treated with ^198^AuNPs GA compared to the control groups. In one of the treated animals, complete tumor regression was achieved. These results demonstrate the effect of the treatment over the 24-day monitoring period.

#### 4.4.2 Biodistribution Assay

Following the treatment period, a biodistribution study was conducted on two animals, as previously defined by the CEUA (Comitê de Ética no Uso de Animais) ethical approval. These preliminary data provide an initial assessment of the dispersion and retention of gum Arabic-coated gold nanoparticles after intratumoral injection in BALB/c Nude mice.

The first animal was evaluated 3 h postinjection, as detailed in Table 2. This early assessment revealed that the nanoparticles had low retention in the tumor (0.11% ID/g), suggesting rapid dispersion from the injection site. A notable uptake in the gallbladder (1.74% ID/g) was observed, indicating potential hepatic processing and excretion *via* the bile. Other organs showed values below 0.5% ID/g, pointing to limited nanoparticle distribution in vital organs. These findings at the 3-h mark highlight the need for optimization of nanoparticle properties or administration strategy to enhance tumor retention and therapeutic efficacy.

**Table 2:** Biodistribution of ^198^AuNPs GA in BALB/c Nude Mice at 3 and 24 h Post-Intratumoral Injection^a^

organ	activity—% DI/g (3 h after injection)	activity—% DI/g (24 h after injection)
Blood	0.13	0.03
Hearth	0.11	0.04
Lungs	0.16	0.04
Liver	0.03	0.37
Kidneys	0.10	0.12
Gallbladder	1.74	0.50
Spleen	0.02	0.68
Stomach	0.15	0.02
Small Intestine	0.01	0.03
Large Intestine	0.03	0.02
Pancreas	0.00	0.06
Bone	0.33	0.04
Muscle	0.01	0.03
Brain	0.01	0.00
Fat	0.00	0.02
Tumor	0.11	0.06
Bladder	0.00	0.01

a The values represent descriptive biodistribution data obtained from two animals (*n* = 1 per time point). The analysis was conducted according to ethical approval by CEUA (protocol 243/19), with a restricted number of animals designated for this assay. No statistical analysis or standard deviation is presented due to the limited sample size.

The biodistribution results at both 3 and 24 h postinjection are presented in Table 2. As observed, there was low tumor retention of the nanoparticles at both time points (0.08% ID/g at 3 h and 0.06% ID/g at 24 h), indicating their rapid dispersion from the injection site. Uptake in the spleen was consistent with clearance by the reticuloendothelial system, increasing from 0.55% ID/g at 3 h to 0.68% ID/g at 24 h. A decrease in uptake in the gallbladder was also observed, from 0.78% ID/g at 3 h to 0.50% ID/g at 24 h, suggesting progressive excretion of the nanoparticles *via* the biliary route. Additionally, the consistently low uptake in other vital organs confirmed the limited systemic distribution of these nanoparticles. These findings emphasize the need for future optimization of the nanoparticle formulation or administration strategy to enhance tumor retention.

In the analysis of biodistribution studies, gold nanoparticles coated with GA and BSA exhibited distinct profiles reflecting their unique interactions with the biological system. ^198^AuNPs GA demonstrated rapid dispersion and varied retention in the studied organs, suggesting different pathways for metabolism and excretion, whereas ^198^AuNPs BSA were characterized by biodistribution patterns that might favor their applicability in certain therapeutic contexts. the complexity of nanoparticle biodistribution and the necessity for specific optimization for each type, aiming to improve retention at the target site and minimize distribution to nontargeted organs. Deepening the understanding of these biodistribution profiles is crucial for the future development of nanoparticles as effective therapeutic vehicles.

#### 4.4.3 Hematological Analysis

The hematological evaluation of animals treated with ^198^AuNPs GA, as presented in Table 3, provides relevant information on the treatment’s tolerability and its impact on the hematological profile. It is important to note that, despite some alterations, the changes in hematological parameters for the treated group remained within the limits typically observed in similar therapeutic interventions, which does not undermine the potential value of the treatment.

**Table 3:** Summary of Key Hematological Indicators of Animals after Administration of ^198^AuNPs GA Treatment^a^,^b^,^c^

treated mice—parameters collected from the complete blood count								
ID animal	μCi dose injected	erythrocytes (6.60–10.0 × 10^6^ / μL)	hemoglobin (14.3–17.3 g/dL)	hematocrit (44% a 51%)	observations red series	leukocytes (4.40–8.30 × 10^3^ μL)	observations white series	platelet count (282 a 481 × 10^3^ μL)
A1	611	6.21 × 10^6^/μL	11.87 g/dL	33%	NCM	7 × 10^3^/μL	NCM	460 × 10^3^/μL
A2	598	6.92 × 10^6^/μL	13.58 g/dL	40%	NCM	15.49x 10^3^/μL	H N + – −	346 × 10^3^/μL
A3	648	4.8 × 10^6^/μL	8.64 g/dL	27%	NCM	32.36 × 10^3^/μL	H N + + −	252 × 10^3^/μL
A6	717	7.13 × 10^6^/μL	13.07 g/dL	43%	NCM	73.20 × 10^3^/μL	H N + + −	298 × 10^3^/μL

a NCM: Normal cell morphology.

b HN: Hypersegmented Neutrophils.

c This table includes values for erythrocytes, hemoglobin, hematocrit, observations on the red series, leukocytes, observations on the white series, and platelet count, with comparisons to the respective reference values. The data were obtained directly from the certified hematological report issued by LAB&VET (São Paulo), without statistical transformation. As standard clinical laboratory values, they are presented in their original format, and no “mean ± SD” or statistical testing was applied.

Unfortunately, the hemogram for animal A4 could not be completed due to sample coagulation, and regrettably, animal A5 passed away before sample collection could take place during the anesthesia period.

Subject Animal 1 (A1) received a dosage of 611 μCi and presented erythrocyte levels slightly below the reference range, suggesting a marginal reduction in red blood cells, yet within expectations. Hemoglobin levels, while lower than the reference range, did not deviate significantly, aligning with the decreased erythrocyte count. The hematocrit levels were below the reference, pointing toward possible anemia, a condition not uncommon in such treatments, though leukocyte and platelet counts remained within normal limits, indicating no severe adverse effects.

Subject A2, dosed with 598 μCi, showed erythrocytes, hemoglobin, and hematocrit levels within the reference range, reflecting the treatment’s nondisruptive nature. However, leukocyte levels were elevated, surpassing the reference range with the presence of hypersegmented neutrophils, hinting at a potential inflammatory response, yet not beyond the scope of typical treatment responses. The platelet count for this subject was also within the expected range, further indicating manageable impact.

For subject A3, who received a dose of 648 μCi (23.98 MBq), the erythrocyte count was below the reference range, indicating anemia, which is within the realm of expected treatment effects. This was further supported by significantly lower hemoglobin and hematocrit levels. Although leukocyte levels were considerably high, suggesting acute inflammation or an immune response, corroborated by hypersegmented neutrophils, such findings are not exceptional for therapeutic trials and fall within manageable ranges. Platelet count was also below the reference range, potentially indicating thrombocytopenia, yet this too can be observed as within treatment expectations.

Subject A6, administered a dose of 717 μCi (26.53 MBq), had erythrocyte, hemoglobin, and hematocrit levels within the reference range, indicating a satisfactory tolerance to the treatment. While leukocytes were extremely elevated, signaling a potent inflammatory or immune response, as confirmed by hypersegmented neutrophils, such responses are not atypical and can be interpreted as a sign of the ’s reaction to the therapeutic agent. Platelet count remained within normal parameters, corroborating the treatment’s manageable hematological impact.

Comparatively, the control group mice, which did not receive any treatment, showcased hematological values within the normal ranges for healthy animals, as outlined in Table 4. This is expected and underscores the integrity of the control group as a baseline. The erythrocyte, hemoglobin, and hematocrit values were consistently within normal reference ranges, and leukocyte counts were consistent with noninflammatory states, further emphasizing the nontreatment-related stability. Platelet counts remained stable, indicating no unusual clotting activity, and confirming the absence of any treatment-related hematological alterations, which is crucial for evaluating the impact of the treatment on the experimental subjects.

**Table 4:** Summary of Key Hematological Indicators for Control Group Animals that Did Not Receive ^198^AuNPs GA Treatment^a^,^b^,^c^

non treated mice—parameters collected from the complete blood count							
ID animal	erythrocytes (6.60–10.0 × 10^6^/μL)	hemoglobin (14.3–17.3 g/dL)	hematocrit (44% a 51%)	observations red series	leukocytes (4.40–8.30 × 10^3^/μL)	observations white series	platelet count (282 a 481 × 10^3^/μL)
A1	7.9 × 10^6^/μL	13.04 g/dL	42%	NCM	4.66 × 10^3^/μL	NCM	284 × 10^3^/μL
A2	8.7 × 10^6^/μL	13.06	40%	NCM	56.80x 10^3^/μL	H N + + −	730 × 10^3^/μL
A3	7.97 × 10^6^/μL	13.4 g/dL	43%	NCM	50.20 × 10^3^/μL	H N + + –	490 × 10^3^/μL
A4	7.39 × 10^6^/μL	12.05 g/dL	40%	NCM	65.20 × 80/ μL	H N + + −	500 × 10^3^/μL
A5	8.31 × 10^6^/μL	14.49 g/dL	43%	NCM	11.46 × 80/ μL	NCM	620 × 10^3^/μL

a NCM: Normal cell morphology.

b HN: Hypersegmented Neutrophils.

c This table presents values for erythrocytes, hemoglobin, hematocrit, leukocytes, and platelet count, compared with their respective reference ranges. The data were obtained directly from the certified hematological report issued by LAB&VET (São Paulo), and are presented in their original format without statistical transformation. As these are individual clinical values, no “mean ± SD” or significance testing was applied

Our observations in the hematological studies revealed that animals treated with ^198^AuNPs GA showed a marginal reduction in red blood cells in some subjects, suggesting a possible mild anemia. Additionally, some animals exhibited elevated leukocyte counts, indicating a potential inflammatory or immune response. These findings highlight the importance of considering not only the direct effects of nanoparticles but also the host biological responses, particularly in animal models with compromised immunity. Even in immunodeficient animals of the BALB/c Nude strain, which were used in our studies, there are residual immune cells, such as macrophages and dendritic cells, that may contribute to inflammatory or immune responses, albeit in a limited capacity.

## 5 Discussion

Nanobrachytherapy with radioactive gold nanoparticles (^198^AuNPs) coated with GA has proven to be a promising strategy for prostate cancer treatment due to its ability to deliver radiation directly to the tumor while minimizing damage to surrounding tissues. Although previous studies by our group explored ^198^AuNPs coated with BSA, the present study focuses exclusively on the GA formulation. References to BSA are included throughout the discussion solely to contextualize the results obtained with GA, and not as part of a direct experimental comparison.^30^

In this study, we explored the application of AuNPs GA for prostate cancer treatment, presenting an innovative synthesis methodology alongside promising biological assay results. This work represents a significant advancement in the field by employing a distinct nanoparticle synthesis approach, characterized by a markedly smaller particle size (approximately 5 nm), as demonstrated through Transmission Electron Microscopy (TEM). This reduced size enhances therapeutic efficacy, optimizes biodistribution, and improves tumor penetration, contributing to the promising results observed in this study.

Ensuring the colloidal stability of AuNPs GA was a central challenge, especially when balancing the concentration-to-activity ratio to maintain an effective therapeutic dose. ζ-Potential measurements and aggregation kinetics (Figures 2 and 3) confirmed that the nanoparticles remain stable over time, despite forming aggregates around 45 nm, as shown by Dynamic Light Scattering (DLS). This behavior is attributed to GA ability to mediate electrostatic interactions and induce steric hindrance. Notably, DLS detected aggregates that are not visible under Transmission Electron Microscopy (TEM), which shows only isolated nanoparticles. The presence of opposite surface charges (approximately ± 107 mV) promotes charge neutralization, while GA’s molecular structure—rich in amine and carboxyl groups—creates steric repulsion, further enhancing colloidal stability.^30^

The stability of the AuNPs GA aggregate solution can be explained by the surface charge distribution of the nanoparticles. The presence of populations with opposite but equivalent surface potentials, as shown in Figure 3, suggests that there are particles with surface charges around ± 107 mV. These nanoparticles aggregate through GA mediation, resulting in charge neutralization. However, nucleation is unlikely, as GA introduces steric hindrance that prevents such processes. Additionally, GA is a polypeptide-based macromolecule capable of forming electrostatic interactions on the AuNP surface through amine and carboxyl functional groups present in its molecular structure. These interactions, combined with its highly branched architecture, induce steric repulsion, further enhancing nanoparticle colloidal stability.^31^

Recognizing the foundational contributions of Dr. Katti and collaborators,^32^ our work builds upon a well-established base of knowledge in nanoparticle synthesis for therapeutic applications. Pioneering studies such as those by Kannan et al.,^31^ Chanda et al.,^33^ and Axial-Bechtel et al.^32^ employed reagents like Tris­(hydroxymethyl)­phosphine, known for their efficiency but associated with higher costs. In contrast, our method introduces a more economical alternative that achieves comparable nanoparticle stability and therapeutic potential, broadening access to nanobrachytherapy strategies. These advancements reinforce the feasibility of cost-effective methodologies, enhancing the accessibility of gold nanoparticle technologies in both research and clinical or industrial applications.

The present findings provide a complementary contribution to existing literature by addressing critical aspects of scalability and sustainability in the synthesis of gold nanoparticles. By proposing an approach that reconciles production efficiency with cost-effectiveness, this work supports the advancement of AuNP-based technologies for broader implementation in therapeutic and diagnostic applications.

The development of more effective and targeted therapies for prostate cancer remains a key challenge in oncology due to the disease’s clinical complexity and resistance to conventional treatments. In this context, the present study investigated the therapeutic potential of radioactive ^198^AuNPs GA, which exhibited selective cytotoxicity toward tumor cells while demonstrating limited impact on surrounding healthy tissues.

Both *in vitro* and *in vivo* assays confirmed the cytotoxic efficacy of ^198^AuNPs GA, particularly in PC-3 cells, which exhibited heightened sensitivity. The nonradioactive form of AuNPs GA was also investigated for comparative purposes, providing a crucial basis for evaluating the differences in mechanisms of action and efficacy between the two formulations. The selection of the PC-3 cell line was driven by its aggressive phenotype and rapid tumor growth, characteristics that not only facilitate efficient data collection but also mimic the heterogeneity of human prostate tumors, making it an ideal model for evaluating targeted therapies

When comparing the results obtained with ^198^AuNPs GA in this study to previously published data from our group on ^198^AuNPs BSA, both formulations have shown cytotoxic effects in tumor cell lines. However, it is important to emphasize that this comparison is contextual, based on prior references, and does not arise from parallel experiments conducted in the present study.

Similarly, the results obtained with ^198^AuNPs GA demonstrated a clear trend of increasing cytotoxicity in cancer cells over time, with exposure duration playing a critical role in amplifying cellular susceptibility. This underscores the need for precise optimization of nanoparticle dosage and delivery specificity to maximize efficacy against tumor cells while mitigating adverse effects on healthy tissues. These considerations are also relevant for other nanoparticle formulations, where selective cytotoxicity and exposure kinetics play a critical role in therapeutic performance.

Contextualizing the results obtained with ^198^AuNPs GA in light of previous data on ^198^AuNPs BSA reinforces the complexity of cellular responses to nanoparticles and highlights the importance of rational nanoparticle design in cancer therapy. While the therapeutic potential of ^198^AuNPs BSA has been well documented, the findings presented here with the GA formulation provide new insights into its efficacy and safety, suggesting promising pathways for targeted and effective treatments in aggressive forms of prostate cancer.

The results of this study demonstrate the biological variability observed among animals treated with ^198^AuNPs GA. One treated animal exhibited complete tumor regression; however, this isolated event may reflect biological variability rather than a reproducible therapeutic effect. While it is an encouraging observation, no clinical relevance should be inferred at this stage, and further studies are required to assess its consistency and significance.

Although the regression observed in one treated animal is anecdotal, similar phenomena have been reported in the literature, even in studies with small sample sizes. For instance, Al-Yasiri et al.^34^ observed significant tumor regression in prostate tumor-bearing mice treated with ^198^AuNPs functionalized with mangiferin, using groups of 5–6 animals. Likewise, Brown et al.^35^ reported complete regression in up to 33% of treated mice using AGuIX-Bi theranostic nanoparticles, in cohorts of 6–9 animals. These examples support the relevance of documenting biological responses in exploratory preclinical studies, particularly when they align with prior research.

Furthermore, ethical and regulatory frameworks, such as the IAEA’s international guidelines (2022),^36^ explicitly acknowledge that early stage radiopharmaceutical studies may ethically use reduced numbers of animals, especially when involving radioactive compounds, provided the design is scientifically justified and aligned with the 3Rs principles (Replacement, Reduction, and Refinement).

This variability may be linked to individual differences in nanoparticle biodistribution, uptake, and metabolism, as well as inherent biological characteristics of the tumors. These results underscore the need for future studies to explore these factors in greater depth, which could provide a more comprehensive understanding of the observed effects. Furthermore, the statistically significant differences observed over time, starting from Day 7, strengthen the therapeutic relevance of ^198^AuNPs GA.

Although further studies with larger sample sizes and more refined methodologies are required, the findings presented here offer a valuable foundation for advancing the investigation of ^198^AuNPs GA as a therapeutic strategy. These initial data support continued preclinical development aimed at optimizing efficacy and safety.

Regarding biodistribution assays, the choice of 3 and 24 h time points was designed to assess both early systemic circulation and delayed tissue retention of the nanoparticles. This dual-time approach facilitates interpretation of therapeutic impact across distinct pharmacokinetic phases and adheres to established preclinical evaluation standards.

These findings emphasize the need for further studies to refine synthesis processes and optimize delivery strategies, aiming to improve nanoparticle targeting and retention in tumor tissues. Moreover, future investigations should explore the molecular mechanisms underlying these therapeutic responses, paving the way for significant advances in the efficacy and safety of ^198^AuNPs GA.

Although limited by the small number of animals, the biodistribution analyses provide preliminary evidence that GA-coated ^198^AuNPs exhibit relatively rapid dispersion from the injection site. These findings are exploratory and descriptive, obtained under strict ethical and logistical constraints due to the use of radioactive material. With only one animal analyzed per time point (*n* = 1), the data lack statistical power and should be interpreted with caution. No statistical tests were applied, and no definitive conclusions should be drawn without validation in larger preclinical studies.

Hematological evaluations provided important information on treatment tolerability. Although we observed some changes in hematological parameters, these changes remained within the limits typically observed in similar therapeutic interventions. This suggests an acceptable safety profile of ^198^AuNPs GA in the context of prostate cancer treatment.

By discussing the results obtained with ^198^AuNPs-GA, this work contributes to the understanding of nanobrachytherapy’s potential in oncology. Prior studies involving BSA coated nanoparticles are referenced only to contextualize the role of surface coatings in therapeutic performance; however, the data presented here refer exclusively to the GA formulation. In addition to emphasizing the relevance of GA based nanoparticles, this study reinforces the importance of continuous innovation in synthesis methods and clinical application of nanomaterials, offering new perspectives for safer and more effective prostate cancer therapies.

## 6 Conclusions

This study represents a significant advancement in the investigation of ^198^AuNPs GA as a promising approach for prostate cancer treatment. Although one animal exhibited complete tumor regression, this result should be considered an anecdotal finding within a limited sample. It does not allow for any inference regarding therapeutic predictability or clinical translation, reinforcing the need for expanded studies. Additionally, the limited sample size (n = 6) restricts the statistical power of the analysis. Nonetheless, the consistent reduction in tumor growth rates suggests a biological trend toward therapeutic efficacy. This modulation of disease progression may represent a clinically relevant benefit, helping to slow tumor progression and improve patient quality of life.

The findings reported here on ^198^AuNPs GA demonstrate excellent colloidal stability, effective tumor growth control, and hematological compatibility, with minimal changes within the expected range for similar therapeutic interventions. Although previous studies by our group with ^198^AuNPs BSA showed relevant therapeutic responses, references to BSA in this manuscript are solely contextual. The discussion of different coatings aims to enrich the understanding of their influence on nanoparticle efficacy and safety, without constituting a direct experimental comparison within the scope of this study.

Given the therapeutic potential of these nanoparticles, future studies should further investigate the differential mechanisms of action between BSA and GA coatings, optimizing their tumor retention and biodistribution. Additionally, the influence of concentration, exposure time, and interactions with different tumor microenvironments should be further explored.

In summary, this study reinforces the relevance of nanobrachytherapy in prostate cancer treatment, providing new perspectives for more effective and targeted approaches. Despite existing challenges, continued research and refinement of ^198^AuNPs GA and separately, of BSA-based formulations could pave the way for safer and more effective therapeutic options for prostate cancer management.
